# Solar ultraviolet-B radiation and vitamin D: a cross-sectional population-based study using data from the 2007 to 2009 Canadian Health Measures Survey

**DOI:** 10.1186/1471-2458-12-660

**Published:** 2012-08-15

**Authors:** Jamie A Greenfield, Philip S Park, Ellie Farahani, Suneil Malik, Reinhold Vieth, Norman A McFarlane, Theodore G Shepherd, Julia A Knight

**Affiliations:** 1 Office of Biotechnology, Genomics and Population Health, Public Health Agency of Canada, 180 Queen Street West, Toronto, Canada; 2 Dalla Lana School of Public Health, University of Toronto, 155 College Street, Toronto, Canada; 3 Samuel Lunenfeld Research Institute, Mount Sinai Hospital, , 60 Murray street, Box 18, Toronto, Canada; 4 Department of Physics, University of Toronto, 60 St. George Street, Toronto, Canada; 5 Centre for EnvironmentUniversity of Toronto, 33 Willcocks Street, Toronto, Canada; 6 Department of Pathology and Laboratory Medicine, Mount Sinai Hospital, 600 University Avenue, Toronto, Canada

**Keywords:** 25-Hydroxyvitamin D, Solar ultraviolet-B irradiance, Public health

## Abstract

**Background:**

Exposure to solar ultraviolet-B (UV-B) radiation is a major source of vitamin D3. Chemistry climate models project decreases in ground-level solar erythemal UV over the current century. It is unclear what impact this will have on vitamin D status at the population level. The purpose of this study was to measure the association between ground-level solar UV-B and serum concentrations of 25-hydroxyvitamin D (25(OH)D) using a secondary analysis of the 2007 to 2009 Canadian Health Measures Survey (CHMS).

**Methods:**

Blood samples collected from individuals aged 12 to 79 years sampled across Canada were analyzed for 25(OH)D (n = 4,398). Solar UV-B irradiance was calculated for the 15 CHMS collection sites using the Tropospheric Ultraviolet and Visible Radiation Model. Multivariable linear regression was used to evaluate the association between 25(OH)D and solar UV-B adjusted for other predictors and to explore effect modification.

**Results:**

Cumulative solar UV-B irradiance averaged over 91 days (91-day UV-B) prior to blood draw correlated significantly with 25(OH)D. Independent of other predictors, a 1 kJ/m^2^ increase in 91-day UV-B was associated with a significant 0.5 nmol/L (95% CI 0.3-0.8) increase in mean 25(OH)D (P = 0.0001). The relationship was stronger among younger individuals and those spending more time outdoors. Based on current projections of decreases in ground-level solar UV-B, we predict less than a 1 nmol/L decrease in mean 25(OH)D for the population.

**Conclusions:**

In Canada, cumulative exposure to ambient solar UV-B has a small but significant association with 25(OH)D concentrations. Public health messages to improve vitamin D status should target safe sun exposure with sunscreen use, and also enhanced dietary and supplemental intake and maintenance of a healthy body weight.

## Background

Climate models predict that in northern middle and high latitudes the expected recovery of stratospheric ozone from the effects of ozone-depleting substances over the coming decades will be substantially augmented by an increase in ozone from climate change [1]. Since stratospheric ozone is a major regulator of solar ultraviolet-B (UV-B) received at the Earth’s surface, this increase in stratospheric ozone implies a decrease in UV-B irradiance. Projected decreases of erythemal UV using the McKinlay-Diffey Erythema action spectrum, which is directly proportional to UV Index, have been estimated between 10 and 15% over the current century [2,3]**.** These atmospheric changes may affect human health since solar UV-B exposure is a major source of vitamin D3. Adequate vitamin D levels are necessary to maintain physiologic calcium and phosphorous for normal bone mineralization and to prevent rickets, osteomalacia and osteoporosis [4]. Vitamin D has also been associated with a decreased risk of infections, cancers, diabetes, cardiovascular disease, and autoimmune diseases [5].

Vitamin D in meaningful amounts is naturally available in only a few foods, such as fatty fish, in addition to fortified milk and supplements [5]. Vitamin D3 is synthesized endogenously in human skin following exposure to UV-B (280–315 nm) radiation in sunlight, which spontaneously photoisomerizes 7-dehydrocholesterol to pre-vitamin D3 [6,7]. Pre-vitamin D3 is subsequently converted to vitamin D3 by thermal isomerisation, which then enters the circulation and is hydroxylated in the liver to long-lived 25-hydroxyvitamin D (25(OH)D3)**.** 25(OH)D is the main indicator of total vitamin D status and represents the sum of vitamin D from both cutaneous synthesis and dietary intake [8].

Since foods are naturally low in vitamin D, the prevalence of vitamin D insufficiency in some populations is thought to result from inadequate exposure to sunlight [8]. The solar zenith angle, which varies by latitude, season and time of day, determines the amount of absorption and scattering of solar UV-B radiation and thus the intensity of sunlight at ground-level [9]**.** Stratospheric ozone and other constituents with absorption features in the UV-B region, clouds, aerosols, surface reflectivity (albedo), and altitude, also affect the amount of solar UV-B reaching the ground [9,10]. The association between solar UV-B and vitamin D is not straightforward, since living in a sunny climate does not ensure sufficient vitamin D status [11,12]. Vitamin D availability also depends on personal and lifestyle factors including skin pigmentation (increased melanin in darker skin naturally blocks cutaneous synthesis of vitamin D3) [13], age (the amount of 7-dehydrocholesterol in the skin decreases with age) [14], dietary and supplemental intake [15], adiposity (storage of vitamin D in adipose tissue decreases its bioavailability) [16], and sunlight exposure (when and how long unprotected skin is exposed) [17].

In Canada, there is a high prevalence of low vitamin D status, particularly in the winter [18-20]. Above 42°N exposure to sunlight during the winter months is not sufficient to initiate cutaneous production of vitamin D3 [21,22]. Approximately 26% of the Canadian population has 25(OH)D concentrations that are inadequate for bone health (< 50 nmol/L) [23]**.** Recent studies in Canada have examined differences in vitamin D status by sex, age, ethnicity, body mass index (BMI), dietary intake, and vitamin D supplementation [23-28]. However, these epidemiologic studies used season, latitude, or time outdoors as proxy measures for solar UV-B exposure. Thus it was not possible to determine the extent to which projected decreases in solar UV-B radiation may impact on 25(OH)D concentrations at the population level. The primary objective of this study was to quantify the association between ground-level solar UV-B irradiance and serum 25(OH)D concentrations in the Canadian population and to examine factors that modify this relationship.

## Methods

### Study population

The 2007 to 2009 CHMS collected physical measurements and blood and urine samples during visits to a mobile clinic at 15 sites across Canada. Demographic and lifestyle information were also gathered through household interviews. Measurements at each site were completed within a period of 36 to 57 days. The target population was household residents aged 6 to 79 years, excluding residents of Indian reserves, Crown lands, certain remote regions, institutions, and full-time members of the Canadian Forces [29]. Approximately 96% of the Canadian population was represented and the overall response rate for participants who completed the mobile clinic visit was 52% at the national level [30]. Blood samples were not collected from respondents with haemophilia or recent chemotherapy treatment (n = 8) and 289 respondents did not provide enough blood for the 25(OH)D assay. Participants less than 12 years of age (n = 908) were excluded from the analysis because lifestyle factors and likely determinants of 25(OH)D differed from adults who are the focus of the current study.

The study sample consisted of 4,398 respondents to the CHMS, representing 26.4 million Canadians aged 12 to 79 years. Informed written consent was obtained from respondents aged 14 years and older [31]. For younger children, a parent or legal guardian provided written consent, and written assent was obtained from the child. Participation was voluntary and respondents could withdraw from any part of the survey at any time. Ethics approval to conduct the survey was obtained from Health Canada’s Research Ethics Board [31]. Data-sharing agreements between Statistics Canada and the Public Health Agency of Canada permitted the use of information collected in the CHMS for statistical and research purposes. Additional information about the CHMS methodology is available in published papers and reports by Statistics Canada [32,33].

### Solar UV-B irradiance

Data on ground-level solar UV-B were calculated using the Tropospheric Ultraviolet and Visible (TUV) Radiation Model (version 4.6) [34]. The TUV radiative transfer model used observations of atmospheric total ozone (O_3_) and total nitrogen dioxide (NO_2_) from the satellite-based Ozone Monitoring Instrument (OMI) and observations of surface albedo from the European Centre for Medium-Range Weather Forecasts (ECMWF) [35]. The shortwave broadband albedo was used due to the difficulty of implementing wavelength-specific values in the TUV model**.** The model was modified to include observations of temperature from the ECMWF and accounted for background aerosols, air density, surface elevation, and solar zenith angle to compute direct and diffuse downwelling components of solar UV-B irradiance at the Earth’s surface every 5 minutes between 11 am and 4 pm at the 15 CHMS sites. UV-B irradiance was integrated over the wavelengths 280 to 315 nm. Three measures of ground-level solar UV-B were examined as predictors of 25(OH)D: (1) daily mean (11 am to 4 pm), (2) daily peak (at local noon) and (3) n-day daily cumulative (11 am to 4 pm) averaged over the 2 to 186 days prior to blood draw.

### Serum 25(OH)D

Serum concentrations of 25(OH)D were analyzed using the LIAISON 25-Hydroxyvitamin D TOTAL assay (Diasorin, Ltd.) in three reference laboratories following standard operating procedures and uniform assay protocols [30]. The lower and upper limits of detection for the chemiluminescent immunoassay are 10 nmol/L and 375 nmol/L, respectively. Analyses were performed singly with an intra and inter-assay variability from 3.2 to 8.5% and 6.9 to 12.7%, respectively. Serum 25(OH)D concentrations less than the lower limit of detection (9.98 nmol/L) were assigned a value half of the lower limit (4.99 nmol/L) [36].

### Other predictors

Sex, age, ethnicity (proxy for skin pigmentation), sun exposure, sunscreen use, vitamin D supplementation, milk consumption, salt water fish consumption, BMI, physical activity index (PAI), cigarette smoking and alcohol use were examined as other predictors of 25(OH)D. PAI was derived in the CHMS from the sum of respondents’ average daily energy expenditures during all leisure time activities in the past three months, and classified as inactive (<1.5 kcal/kg/day), moderately active (1.5 to 2.9 kcal/kg/day), or active (≥ 3.0 kcal/kg/day) [30]. Information about these predictors was collected in the CHMS in-home interview questionnaire, clinic questionnaire or mobile clinic visit [30]. 

### Statistical analyses

A square root transformation was applied to normalize the 25(OH)D distribution. Simple linear regression was performed for each predictor of 25(OH)D. Individual measures of solar UV-B irradiance were bimodally distributed due to the timing and location of data collection, with each CHMS site sampled within a three month period. Measures of solar UV-B irradiance were modelled both as categorical and continuous variables and a second-order term was added to the model to allow a curvilinear relationship with 25(OH)D. There was no evidence of non-linearity and results were similar for categorical and continuous measures of solar UV-B irradiance. Data are presented for the continuous measure only. The other predictors were examined as categorical variables to explore interactions with solar UV-B irradiance and to perform subgroup analyses. Levels of the variables measured in the CHMS were collapsed to minimize the degrees of freedom while ensuring an adequate number of observations within each subgroup.

A multivariable model was built by backwards stepwise selection from the full model and included all significant predictors. Sunscreen use, season and latitude were not adjusted for in the multivariable model because information about sunscreen use was collected only for participants who reported at least 30 minutes of sun exposure on a typical weekend or day off in the summer, and season and latitude are collinearly related to solar UV-B irradiance. Effect modification of the relationship between solar UV-B irradiance and 25(OH)D was examined by simultaneously testing interaction terms in the full model. Subgroup analyses were performed within levels of the significant effect modifiers. Point estimates were calculated using the survey weights to be representative of the Canadian population covered and to adjust for non-response. Variance estimates were calculated using the bootstrap method to account for the complex sampling design of the CHMS. Confidence intervals (CIs) and P-values were calculated using 11 degrees of freedom to reflect the 15 collection sites (clusters) and five regions (strata) [30]. Significance was defined as P<0.05 for main effects and P<0.01 for interaction effects. The multivariable model included data for 4,354 study participants who had non-missing observations for all independent predictors. Statistical analyses were performed using SAS (version 9.2).

## Results

Descriptive statistics for the study participants are presented in Table 1. The mean concentration of 25(OH)D weighted for the Canadian population aged 12 to 79 years was 67.2 nmol/L (95% CI 64.9-69.6) and ranged from 5.0 to 274.6 nmol/L. In simple linear regression, measures of solar UV-B irradiance were positively correlated with square root transformed 25(OH)D, although the relationship was non-significant for daily mean and daily peak solar UV-B (data not shown). The correlation between n-day daily cumulative solar UV-B and 25(OH)D was greatest for the 91-days prior to blood draw (91-day UV-B) (r^2^ = 0.03) (Table 2). The mean 91-day UV-B weighted for the Canadian population was 12.7 kJ/m^2^ (95% CI 9.9-15.5) and ranged from 1.4 to 24.8 kJ/m^2^. The seasonal variation in UV-B exposure as shown by the average daily UV-B (and range) calculated from the model output for the 15 CHMS sites over the two years of data collection was 4.4 kJ/m^2^ (1.0 to 15.7 kJ/m^2^) in the winter (January to March); 19.5 kJ/m^2^ (9.3 to 27.3 kJ/m^2^) in the spring (April to June); 19.5 kJ/m^2^ (11.3 to 26.5 kJ/m^2^) in the summer (July to September); and 5.4 kJ/m^2^ (1.6 to 14.8 kJ/m^2^) in the fall (October to December). In univariate analysis, higher mean 25(OH)D was associated with female sex, older age (60–79 years), white ethnicity, longer duration of sun exposure (≥ 30 min/d), more frequent sunscreen use (sometimes, often or always), vitamin D supplementation, increased milk consumption (≥ once/day), lower BMI (< 30 kg/m^2^), increased PAI (active or moderately active), current alcohol use, blood draw in the summer (July to September) compared to the winter (December to February), and medium latitude (45–47°N) compared to high latitude (49–54°N) (Table 2).

**Table 1:** Characteristics of the sample and household population aged 12 to 79 years, Canada, 2007 to 2009

**Variable**	**Sample (Unweighted)**	**Population (Weighted)**
	**n**	**% (95% CI)**
Sex		
Female	2289	50.0 (49.2, 50.7)
Male	2109	50.0 (49.6, 50.5)
Age		
12-19 years	950	12.0 (11.6, 12.4)
20-59 years	2375	69.7 (69.1, 70.4)
60-79 years	1073	18.3 (18.0, 18.5)
Ethnicity		
White	3696	81.7 (74.4, 88.8)
Non-White	696	18.3 (11.0, 25.5)
Sun Exposure		
< 30 min/d	833	19.2 (16.1, 22.2)
≥ 30 min/d and < 2 h/d	1210	27.5 (24.5, 30.4)
≥ 2 h/d	2350	53.4 (48.6, 58.0)
Sunscreen Use*		
Never or rarely	1754	49.3 (45.4, 53.0)
Sometimes	672	18.9 (16.9, 21.0)
Often or always	1134	31.9 (28.7, 35.0)
Vitamin D Supplementation		
No	3826	85.1 (78.8, 88.5)
Yes	572	14.9 (12.4, 20.4)
Milk Consumption		
< Once/d	1790	42.9 (40.7, 45.2)
Once/d	1425	33.7 (32.5, 34.9)
> Once/d	1183	23.4 (21.1, 25.8)
Salt Water Fish Consumption		
< Once/week	2178	49.7 (45.9, 53.5)
Once/week	1164	25.8 (24.0, 27.6)
> Once/week	1056	24.5 (21.5, 27.5)
Body Mass Index		
< 25 kg/m^2^	1930	42.3 (37.7, 46.6)
≥ 25 and < 30 kg/m^2^	1453	34.7 (31.5, 37.2)
≥ 30 kg/m^2^	983	23.0 (19.6, 25.9)
Physical Activity Index		
Inactive	2120	51.8 (46.3, 57.4)
Moderately Active	1107	24.5 (21.8, 27.1)
Active	1170	23.7 (20.1, 27.3)
Cigarette Smoking		
Non-Smoker	3605	80.0 (77.0, 82.2)
Current Smoker	792	20.0 (18.1, 22.7)
Alcohol Use		
Non-Drinker	1007	18.0 (16.4, 20.3)
Current Drinker	3390	82.0 (79.9, 83.4)
Season		
January to March	896	21.8^F^ (0.0, 43.6)
April to June	1269	31.4^F^ (4.7, 58.1)
July to September	1063	20.5^F^ (5.8, 35.2)
October to December	1170	26.3^F^ (8.0, 44.6)
Latitude		
43-44 ^o^N	1615	38.8 (37.8, 39.8)
45-47 ^o^N	1548	30.9 (30.7, 31.1)
49-54 ^o^N	1235	30.3 (29.8, 30.8)

*Sunscreen use was collected only for participants who reported ≥ 30 min/d sun exposure.

^F^Interpret with caution (coefficient of variation 16.6-33.3%).

**Table 2:** Simple linear regression for square root transformed 25(OH)D weighted for the household population aged 12 to 79 years, Canada, 2007 to 2009

**Variable**	β* ± SE	25(OH)D**	**P**	**Model**	
		**(95% CI)**		**r**^**2**^	
91-Day UV-B (kJ /m^2^)	0.04 ± 0.01	0.6 (0.2, 0.9)	0.0002	0.03	
Sex				0.01	
Female	(Reference)	67.1 (65.0, 69.1)			
Male	−0.30 ± 0.07	62.3 (60.1, 64.4)	< 0.0001		
Age				0.01	
12-19 years	0.14 ± 0.10	65.4 (62.3, 68.5)	0.16		
20-59 years	(Reference)	63.2 (60.6, 65.9)			
60-79 years	0.41 ± 0.10	69.8 (66.7, 73.1)	< 0.0001		
Ethnicity				0.09	
White	(Reference)	68.4 (64.3, 72.8)			
Non-White	−1.27 ± 0.13	49.0 (46.0, 52.1)	< 0.0001		
Sun Exposure				0.01	
< 30 min/d	(Reference)	58.9 (55.9, 61.9)			
≥ 30 min/d and < 2 h/d	0.34 ± 0.10*	64.2 (61.2, 67.2)	0.0003		
≥ 2 h/d	0.52 ± 0.09	67.0 (64.4, 69.7)	< 0.0001		
Sunscreen Use				0.01	
Never or rarely	(Reference)	64.1 (61.6, 66.6)			
Sometimes	0.11 ± 0.07	65.9 (63.8, 68.1)	0.09		
Often or always	0.32 ± 0.07	69.3 (67.0, 71.6)	< 0.0001		
Vitamin D Supplementation				0.01	
No	(Reference)	63.6 (61.1, 66.1)			
Yes	0.41 ± 0.13	70.2 (66.0, 74.6)	0.002		
Milk Consumption				0.03	
< Once/d	(Reference)	60.0 (57.8, 62.2)			
Once/d	0.36 ± 0.06^**^	65.7 (63.9, 67.6)	< 0.0001		
> Once/d	0.75 ± 0.06	72.1 (70.1, 74.1)	< 0.0001		
Salt Water Fish Consumption				< 0.01	
< Once/week	(Reference)	63.8 (60.9, 66.8)			
Once/week	0.04 ± 0.08	64.5 (62.0, 67.0)	0.60		
> Once/week	0.17 ± 0.16	66.6 (61.7, 71.7)	0.27		
Body Mass Index				0.02	
< 25 kg/m^2^	(Reference)	67.2 (64.1, 70.5)			
≥ 25 and < 30 kg/m^2^	−0.07 ± 0.08^#^	66.1 (63.7, 68.6)	0.38		
≥ 30 kg/m^2^	−0.57 ± 0.09	58.2 (55.6, 60.8)	< 0.0001		
Physical Activity Index				0.02	
Inactive	(Reference)	60.8 (58.3, 63.4)			
Moderately Active	0.46 ± 0.07	68.2 (65.9, 70.6)	< 0.0001		
Active	0.54 ± 0.11	69.6 (66.1, 73.2)	< 0.0001		
Cigarette Smoking				< 0.01	
Non-Smoker	(Reference)	65.3 (63.0, 67.6)			
Current Smoker	−0.19 ± 0.12	62.3 (58.8, 65.9)	0.10		
Alcohol Use				0.01	
Non-Drinker	(Reference)	60.4 (56.7, 64.2)			
Current Drinker	0.33 ± 0.10	65.6 (62.6, 68.7)	0.0006		
Season				0.03	
January to March	(Reference)	58.6 (48.1, 70.0)			
April to June	0.36 ± 0.41	64.2 (51.9, 77.8)	0.38		
July to September	0.85 ± 0.42	72.2 (58.9, 87.0)	0.04		
October to December	0.38 ± 0.39	64.6 (52.8, 77.5)	0.33		
Latitude				0.02	
43-44^o^N	(Reference)	63.0 (59.3, 66.7)			
45-47^o^N	0.43 ± 0.24^##^	70.0 (62.4, 78.1)	0.07		
49-54^o^N	−0.09 ± 0.12	61.5 (57.8, 65.3)	0.44		

*For 91-day UV-B, the estimated mean increase in square root transformed 25(OH)D for a 1 kJ/m^2^ increase in solar UV-B irradiance between 11 am and 4 pm at the CHMS collection sites averaged over the 91-days prior to blood draw; for other predictors, the estimated mean change in square root transformed 25(OH)D compared to the reference category.

**For 91-day UV-B, the estimated mean increase in back transformed 25(OH)D (nmol/L) for a 1 kJ/m^2^ increase in solar UV-B irradiance between 11 am and 4 pm at the CHMS collection sites averaged over the 91-days prior to blood draw; for other predictors,, the estimated mean change in back transformed 25(OH)D (nmol/L) compared to the reference category.

^*^Significantly different than ≥ 2 h/d (P = 0.004).

^**^Significantly different than > once/d (P<0.0001).

^#^Significantly different than ≥ 30 kg/m^2^ (P<0.0001).

^##^Significantly different than 49–54^o^N (P = 0.01).

Significant main effects in the multivariable model were 91-day UV-B, sex, age, ethnicity, sun exposure, vitamin D supplementation, milk consumption, BMI and PAI (P<0.05) (Table 3). The multivariable model explained 21% of the total variability in 25(OH)D. Ethnicity was the strongest predictor of 25(OH)D and accounted for 6% of the total variability, followed by 91-day UV-B (3%), BMI (2%) and milk consumption (2%). Vitamin D supplementation, sex, age, sun exposure, and PAI each accounted for approximately 1% of the total variability in 25(OH)D. Adjusted for personal and lifestyle factors, a 1 kJ/m^2^ increase in 91-day UV-B was associated with a significant 0.5 nmol/L (95% CI 0.3-0.8) increase in mean 25(OH)D (P = 0.0001). Interactions between 91-day UV-B and age (P<0.0001) and PAI (P = 0.002) were significant. The magnitude of the association between 91-day UV-B and 25(OH)D was approximately two times greater within the age group 12 to 19 years (β = 0.07) and within the physically active group (β = 0.06), compared to the other subgroups (Table 4). There was no significant association between 91-day UV-B and 25(OH)D within the age group 60 to 79 years (P = 0.54).

**Table 3:** Multivariable linear regression for square root transformed 25(OH)D weighted for the household population aged 12 to 79 years, Canada, 2007 to 2009

**Variable**	β* ± SE	Mean 25(OH)D**	**P**	**Partial**
		**(95% CI)**		**r**^2^***
91-Day UV-B	0.04 ± 0.01	0.5 (0.3, 0.8)	0.0001	0.03
Sex				0.01
Female	(Reference)	54.2 (49.6, 58.9)		
Male	−0.31 ± 0.05	49.8 (48.3, 51.3)	< 0.0001	
Age				0.01
12-19 years	−0.13 ± 0.10	52.3 (49.6, 55.0)	0.16	
20-59 years	(Reference)	54.2 (49.6, 58.9)		
60-79 years	0.36 ± 0.09	59.6 (56.8, 62.4)	0.0001	
Ethnicity				0.06
White	(Reference)	54.2 (49.6, 58.9)		
Non-White	−1.10 ± 0.12	39.3 (36.5, 42.1)	< 0.0001	
Sun Exposure				0.01
< 30 min/d	(Reference)	54.2 (49.6, 58.9)		
≥ 30 min/d and < 2 h/d	0.22 ± 0.07^*^	57.5 (55.4, 59.6)	0.002	
≥ 2 h/d	0.42 ± 0.09	60.5 (57.7, 63.4)	< 0.0001	
Vitamin D Supplementation				0.01
No	(Reference)	54.2 (49.6, 58.9)		
Yes	0.44 ± 0.06	60.8 (59.1, 62.6)	< 0.0001	
Milk Consumption				0.02
< Once/d	(Reference)	54.2 (49.6, 58.9)		
Once/d	0.29 ± 0.05^**^	58.6 (57.0, 60.2)	< 0.0001	
> Once/d	0.58 ± 0.06	63.1 (61.3, 64.9)	< 0.0001	
Body Mass Index				0.02
< 25 kg/m^2^	(Reference)	54.2 (49.6, 58.9)		
≥ 25 and < 30 kg/m^2^	−0.13 ± 0.07^#^	52.2 (50.3, 54.2)	0.05	
≥ 30 kg/m^2^	−0.63 ± 0.05	45.3 (44.0, 46.7)	< 0.0001	
Physical Activity Index				0.01
Inactive	(Reference)	54.2 (49.6, 58.9)		
Moderately Active	0.30 ± 0.08	57.1 (55.4, 58.8)	0.0009	
Active	0.19 ± 0.06	58.6 (56.3, 61.1)	0.0002	

Model r^2^ = 0.21.

*For 91-day UV-B, the estimated mean increase in square root transformed 25(OH)D for a 1 kJ/m^2^ increase in solar UV-B irradiance between 11 am and 4 pm at the CHMS collection sites averaged over the 91-days prior to blood draw; for other predictors, the estimated mean change in square root transformed 25(OH)D compared to the reference category.

**For 9-day UV-B, the estimated mean increase in back transformed 25(OH)D (nmol/L) for a 1 kJ/m^2^ increase in solar UV-B irradiance between 11 am and 4 pm at the CHMS collection sites averaged over the 91-days prior to blood draw; for other predictors, the estimated mean change in back transformed 25(OH)D (nmol/L) compared to the reference category.

***Total variance in square root transformed 25(OH)D explained by each predictor, controlling for all other variables.

^*^Significantly different than ≥ 2 h/d (P = 0.003).

^**^Significantly different than > once/d (P<0.0001).

^#^Significantly different than ≥ 30 kg/m^2^ (P<0.0001).

**Table 4:** Multivariable linear regression model for 91-day UV-B and square root transformed 25(OH)D within subgroups of age and physical activity index, weighted for the household population aged 12 to 79 years, Canada, 2007 to 2009

**Effect Modifier**	β* ± SE	Mean 25(OH)D**	**P**	**Model**	**Partial**
		**(95% CI)**		**r**^**2**^	**r**^2^***
Age					
12-19 years	0.07 ± 0.01	0.9 (0.6, 1.3)	< 0.0001	0.27	0.07
20-59 years	0.04 ± 0.01	0.6 (0.3, 0.9)	0.0001	0.22	0.03
60-79 years	−0.01 ± 0.01	−0.1 (−0.5, 0.2)	0.54	0.09	<0.01
Physical Activity Index					
Inactive	0.03 ± 0.01	0.5 (0.0, 0.8)	0.0009	0.20	0.02
Moderately Active	0.03 ± 0.01	0.4 (0.1, 0.8)	0.02	0.19	0.01
Active	0.06 ± 0.02	0.9 (0.4, 1.3)	0.0001	0.19	0.07

Adjusted for sex, age, ethnicity, sun exposure, vitamin D supplementation, milk consumption, BMI, and PAI.

*Estimated mean increase in square root transformed 25(OH)D for a 1 kJ/m^2^ increase in solar UV-B irradiance between 11 am and 4 pm at the CHMS collection sites averaged over the 91-days prior to blood draw (91-day UV-B).

**Estimated mean change in back transformed 25(OH)D (nmol/L) for a 1 kJ/m^2^ increase in solar UV-B irradiance between 11 am and 4 pm for the CHMS collection sites averaged over the 91-days prior to blood draw (91-day UV-B).

***Total variance in square root transformed 25(OH)D explained by 91-day UV-B, controlling for all other variables.

## Discussion

We used a secondary analysis of data from the 2007 to 2009 CHMS to examine the association between serum concentrations of 25(OH)D and ground-level solar UV-B. This is the first study in Canada to quantify the association between ambient solar UV-B and 25(OH)D in a nationally representative sample. The comprehensiveness of the CHMS made it possible to adjust for personal and lifestyle factors that influence vitamin D status and to examine effect modification. There is currently no standardized method to assess sunlight exposure to explain variation in vitamin D status. The time period over which solar UV-B exposure is measured varies markedly across studies, from one week up to one year. In our study, 25(OH)D levels measured on a given date were influenced the most by the cumulative effect of ambient solar UV-B radiation over the 91-days prior to blood draw. The most relevant time period for solar UV-B exposure with respect to 25(OH)D has not been addressed in previous studies. Unadjusted for other factors, season was a relatively good proxy for 91-day UV-B compared to latitude. Significant differences in 25(OH)D were evident only between the summer (July to September) and winter (January to March) and between high (49–54°N) and medium (45–47°N) latitudes. However, geographic heterogeneity across the CHMS sites may be too small to capture the effect of latitude. Additionally, it is difficult to separate season and latitude effects due to the sampling design of the CHMS.

In a recent study from the Women’s Health Initiative Calcium plus Vitamin D Clinical Trial, mean annual regional solar irradiance at the location of residence accounted for 1% of the total variability in 25(OH)D, whereas month of blood draw accounted for 3% [37]. Compared to our results, this suggests that the one year period over which solar irradiance was averaged was too long to accurately capture the effect of solar UV-B exposure. Another population-based study used data from the Adventist Health Study-2 to examine erythemal zone (average monthly noon erythemal radiation at the location of residence) during the two months prior to blood collection, UV season (categorized into three groups according to erythemal zone), season, and latitude as predictors of 25(OH)D [38]. In multivariable analysis, UV season and erythemal zone were more strongly associated with 25(OH)D than season and latitude, demonstrating that measures of solar UV irradiance, as opposed to season and latitude as proxies, are better predictors of 25(OH)D. Our results demonstrate that future epidemiologic studies should assess solar UV-B exposure over a three month period to best capture the variability in 25(OH)D concentrations.

Similar to our results, the population-based Canadian Multicentre Osteoporosis Study identified fall and winter season, BMI ≥ 30 kg/m^2^, darker skin pigmentation, and lower vitamin D supplementation as the strongest predictors of decreased 25(OH)D among Canadians over 35 years of age across seven cities [25]. Age was not found to be a significant independent predictor of 25(OH)D; however, most study participants were older than 51 years of age. Regular participation in physical activity was a significant predictor for females only. There was a significant interaction between vitamin D supplementation and season, which was not found in our study; although, the dose of vitamin D supplementation for respondents to the CHMS was not measured. In our study, we found that age and PAI were significant effect modifiers of the relationship between 91-day UV-B and 25(OH)D. The interaction with age may reflect that synthesis of vitamin D3 decreases with increasing age due to reduced concentrations of 7-dehydrocholesterol in the skin as well as alterations in skin morphology [14]. Although the association between 91-day UV-B and 25(OH)D was strongest within the youngest age group, the oldest age group had the highest levels of 25(OH)D. This may suggest that dietary and supplemental intake of vitamin D play an important role in achieving adequate levels of 25(OH)D among older individuals. The interaction with PAI may suggest that physical activity is a good proxy for time spent outdoors in the sun. This is consistent with results from the Third National Health and Nutrition Examination Survey, in which regular outdoor physical activity, as opposed to intense indoor physical activity, was associated with higher levels of 25(OH)D [39].

We estimate that a 10 to 15% decrease in solar erythemal UV projected over the current century [2,3] corresponding to a decrease in solar UV-B irradiance of less than 2 kJ/m^2^ in Canada, would be associated with less than a 1 nmol/L decrease in mean 25(OH)D for the population. Although solar UV-B irradiance is significantly associated with 25(OH)D concentrations, the small magnitude of effect may be due to inadequate sun exposure at the individual level as a result of behaviour and/or the “vitamin D winter” that is characteristic of high latitudes. Public health messages should increase awareness about practising safe sun exposure optimal for vitamin D3 synthesis during the summer in addition to promoting dietary and supplemental intake of vitamin D and proper nutrition and physical activity to maintain a healthy body weight. Vitamin D reference intakes should be set at levels high enough to prevent vitamin D insufficiency among individuals who do not obtain adequate solar UV-B exposure.

The main strengths of our study include its large sample size, which was representative of the Canadian population, and the low frequency of missing data. In contrast to most epidemiologic studies, we did not use season or latitude as a proxy for solar UV-B exposure, and we were able to examine personal and lifestyle factors that influence vitamin D status. A limitation of our study is the low response rate for blood draw among the CHMS respondents. Measurement error associated with solar UV-B irradiance, serum 25(OH)D concentrations, and other predictors likely contribute to the low variability in 25(OH)D captured in the multivariable regression model. Solar UV-B irradiances were calculated for clear-sky conditions because of the highly variable and unpredictable effect of clouds on solar UV-B irradiance [9]. The ECMWF cloud field does not contain cloud base and cloud top heights, which are required in the TUV model. The adjustment for background aerosols did not account for highly polluted regions, which may reduce ground-level solar UV-B due to scattering and absorption [9]. Lastly, solar UV-B irradiances calculated using the TUV model were not weighted for the vitamin D action spectrum, which corresponds to the conversion of 7-dehydrocholesterol to pre-vitamin D3**.** Limitations of the CHMS data include a lack of assessment of percent fat, skin pigmentation, the duration or timing of recent sun exposure, sunscreen use in all participants, typical clothing coverage outdoors, recent travel to a sunny climate, and the frequency or dose of vitamin D supplementation. Despite these limitations, our results are comparable to recent predictive models that explained 21 to 42% of the total variability in 25(OH)D [37,38,40-42]. Additional factors not accounted for, such as genetic differences in vitamin D related genes, may play an important role in determining 25(OH)D concentrations [43,44]. It is likely that many factors each impart a small but significant influence on the vitamin D status of human populations.

## Conclusions

Solar UV-B irradiance explains a small but statistically significant proportion of the total variability in 25(OH)D concentrations. Future climate change and ozone recovery is expected to have a small effect on mean serum 25(OH)D for the Canadian population, at latitudes 43–54°N, suggesting that public health messages and interventions to promote sufficient vitamin D status should target behavioural factors including safe sun exposure with sunscreen use, enhanced dietary and supplemental intake of vitamin D and maintenance of a healthy body weight. These results likely pertain to other populations in developed countries at similar latitudes. Future investigations using longitudinal studies and observations of UV-B irradiance are needed to better evaluate the causal relationship between ambient solar UV-B and vitamin D status in humans.

## Abbreviations

β: Beta coefficient; 25(OH)D: 25-hydroxyvitamin D; 91-day: UV-B cumulative solar ultraviolet-B irradiance averaged over 91-days; BMI: Body mass index; CHMS: Canadian Health Measures Survey CI Confidence interval; P: P-value; PAI: Physical activity index; r^2^: Coefficient of determination; TUV: Tropospheric Ultraviolet and Visible; UV: Ultraviolet; UV-B: 280–315 nm.

## Competing interests

The authors declare that they have no competing interests.

## Authors’ contributions

RV, SM, JAK, TGS, EF and NAM participated in the conception and design of the study, coordination, and its final approval. PSP performed the solar UV-B irradiance calculations. JAG performed the statistical analysis and drafted the manuscript. JAK, TGS, SM, EF, NAM, and PSP revised the manuscript for important intellectual content. All authors read and approved the final manuscript.

## Pre-publication history

The pre-publication history for this paper can be accessed here:

http://www.biomedcentral.com/1471-2458/12/660/prepub
